# Fatal Case of Rupture of the Spleen

**Published:** 1844-08

**Authors:** Wm. B. Herrick


*Fatal Case of Rupture of the Spleen.* By Wm. B. Herrick, M.D.

In March last, I was called by a coroner to examine the body of J. W., a man of about 40 years of age, robust and muscular, who had previously shown no very marked indications of permanent bad health.

It appeared, from evidence before the coroner's jury, that the deceased had died soon after being engaged in a scuffle with B. R., (a man of about his own size and physical development); that J. W. had received one blow, from the fist of his opponent, upon the left hypocondrical region; that after this, the combatants had clinched each other, and so equal were their exertions for five or ten minutes, that it seemed doubtful which would come off victor; at length, however, the strength of J. W. seemed suddenly to fail. He turned pale, staggered, and sunk helpless upon the ground, complaining of nausea, faintness, and pain in the left side. He was carried, in a sinking condition, a short distance to a house, where he expired, in about fifteen minutes after the termination of the conflict.

A hasty examination of the body, twenty-four hours after death, showed no marks of violence upon the exterior. The cavity of the pericardium contained about two ounces of effused serum. In other respects the contents of the thorax appeared normal. But upon cutting through the abdominal parietes, exit was given to between two and three quarts of dark, partially coagulated blood.

An extended incision brought into view the spleen, enlarged to about five times its natural dimensions, and so soft in texture as to be easily broken down under slight pressure from a finger. Upon its posterior surface was a lacerated fissure of about five inches in length, extending deep into the centre of the organ. Evidently it was from the divided blood vessels of this torn structure, that internal hemorrhage had taken place to such an extent as to cause immediate death.

The coroners verdict was as follows: "Death from lacerated diseased spleen, caused by a blow, fall, or over exertion, while engaged in a scuffle with B. R." B. R. was tried for manslaughter, and acquitted by the Circuit Court.

From the history of the above case, we learn how very important it is to have thorough port mortem examinations, in many alleged cases, of manslaughter and murder. Without the information gained by examination, it is evident that even an enlightened jury might, under similar circumstances, feel bound to bring in a verdict against an unoffending and innocent man.

